# 5-(2-Cyano­benz­yl)-4,5,6,7-tetra­hydro­thieno[3,2-*c*]pyridin-2-yl acetate

**DOI:** 10.1107/S1600536813009513

**Published:** 2013-04-13

**Authors:** Xiao-Shuai Xie, Shuai Mu, Ying Liu, Deng-Ke Liu

**Affiliations:** a Tianjin First Central Hospital, Tianjin 300192, People’s Republic of China; b Tianjin Medical University, Tianjin 300070, People’s Republic of China; c School of Chemical Engineering and Technology, Tianjin University, Tianjin 300072, People’s Republic of China; d Tianjin Institute of Pharmaceutical Research, Tianjin 300193, People’s Republic of China

## Abstract

In the title mol­ecule, C_17_H_16_N_2_O_2_S, the tetra­hydro­pyridine ring exhibits a half-chair conformation. The mean planes of the ester chain and benzene ring are twisted by 5.5 (1) and 81.32 (5)°, respectively, from the plane of thio­phene ring. In the crystal, weak C—H⋯O inter­actions link mol­ecules related by translation along [100] into chains.

## Related literature

For the crystal structures of related compounds, see: Wang *et al.* (2010 ▶); Yang *et al.* (2012 ▶). For details of the synthesis, see: Zhou *et al.* (2011 ▶).
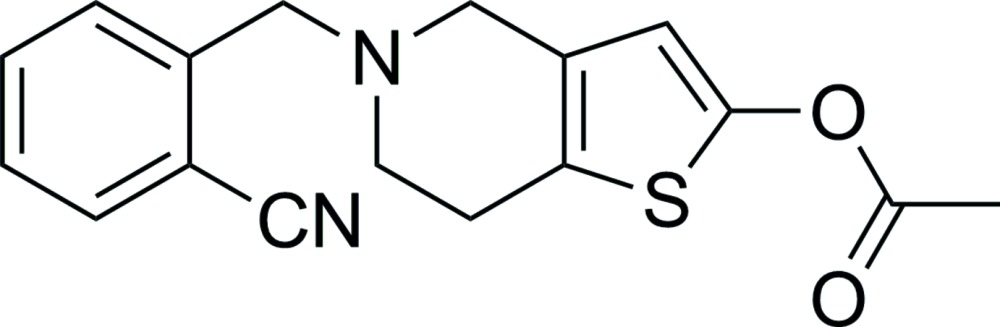


## Experimental

### 

#### Crystal data

C_17_H_16_N_2_O_2_S
*M* *_r_* = 312.38
Monoclinic, 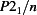

*a* = 14.174 (3) Å
*b* = 5.9321 (12) Å
*c* = 18.796 (4) Å
β = 99.06 (3)°
*V* = 1560.7 (5) Å^3^
*Z* = 4
Cu *K*α radiation
μ = 1.91 mm^−1^
*T* = 113 K
0.26 × 0.24 × 0.22 mm

#### Data collection

Rigaku Saturn diffractometer
Absorption correction: multi-scan (*CrystalClear*; Rigaku/MSC, 2005 ▶) *T*_min_ = 0.636, *T*_max_ = 0.678
16000 measured reflections
3034 independent reflections
2819 reflections with *I* > 2σ(*I*)
*R*_int_ = 0.045

#### Refinement

*R*[*F* ^2^ > 2σ(*F* ^2^)] = 0.038
*wR*(*F* ^2^) = 0.098
*S* = 1.08
3034 reflections
201 parameters
1 restraint
H-atom parameters constrained
Δρ_max_ = 0.30 e Å^−3^
Δρ_min_ = −0.28 e Å^−3^

### 

Data collection: *CrystalClear* (Rigaku/MSC, 2005 ▶); cell refinement: *CrystalClear*; data reduction: *CrystalClear*; program(s) used to solve structure: *SHELXS97* (Sheldrick, 2008 ▶); program(s) used to refine structure: *SHELXL97* (Sheldrick, 2008 ▶); molecular graphics: *SHELXTL* (Sheldrick, 2008 ▶); software used to prepare material for publication: *CrystalStructure* (Rigaku/MSC, 2005 ▶).

## Supplementary Material

Crystal structure: contains datablock(s) global, I. DOI: 10.1107/S1600536813009513/cv5399sup1.cif

Structure factors: contains datablock(s) I. DOI: 10.1107/S1600536813009513/cv5399Isup2.hkl

Supplementary material file. DOI: 10.1107/S1600536813009513/cv5399Isup3.cdx

Supplementary material file. DOI: 10.1107/S1600536813009513/cv5399Isup4.cml

Additional supplementary materials:  crystallographic information; 3D view; checkCIF report

## Figures and Tables

**Table 1:** Hydrogen-bond geometry (Å, °)

*D*—H⋯*A*	*D*—H	H⋯*A*	*D*⋯*A*	*D*—H⋯*A*
C11—H11⋯O2^i^	0.95	2.53	3.3346 (19)	143

Symmetry code: (i) 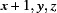.

**Fig. 1.**
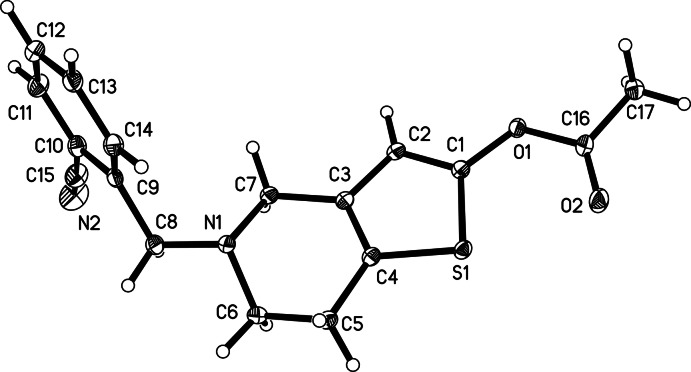
The molecular structure of (I) showing the atom-numbering scheme and 50% probability displacement ellipsoids.

## supplementary crystallographic information

### Comment

As a continuation of our structural study of tetrahydrothienopyridine derivatives (Yang *et al.*, 2012), herein we present the crystal structure of the title compound (I).

In (I) (Fig. 1), all bond lengths and angles are normal and correspond to those observed in the related compounds 5-[(2-cyclopropylcarbonyl)(2- fluorophenyl)methyl]-4,5,6,7-tetrahydrothieno[3,2-*c*]pyridin- 2-yl acetate (Prasugrel) (Wang *et al.*, 2010) and 5-(2-chlorobenzyl)-4,5,6,7-tetrahydrothieno[3,2-*c*]pyridin-2-yl acetate (Yang *et al.*, 2012). The ester chain in (I) is almost planar with a mean deviation of 0.0021 Å. The tetrahydropyridine ring exhibits a half-chair conformation. The mean planes of the ester chain and benzene ring are twisted at 5.5 (1) and 81.32 (5)°, respectively, from the plane of thiophene ring. In the crystal, weak C—H···O interactions (Table 1) link the molecules related by translation in [100] into chains.

### Experimental

The title compound was prepared according to the method of Zhou *et al.* (2011). 19.2 g of 5,6,7,7a-tetrahydrothieno[3,2-*c*]pyridin-2(4*H*)-one hydrochloride and 29 g of *N*-methyl morpholine were dissolved in 100 ml of CHCl_3_. 42.6 g of 2-(bromomethyl)benzonitrile was dropwised into the mixture and then refluxed for 4 h. After filtration, the resulting filtrate was evaporated under reduced pressure. The residue was dissolved in diethyl ether, adjust the pH=5 to get 2-{(2-oxo-7,7a-dihydrothieno [3,2-*c*]pyridin-5(2*H*,4*H*,6*H*)-yl)methyl} benzonitrile as an intermediate. The intermediate, together with 14.5 g of *N*-methyl morpholine and 10 g of acetic anhydride was dissolved in 150 ml of acetonitrile and stirred under 30°C for 2 h. The mixture was evaporated under reduced pressure and yellow oil was obtained. The oil was dissolved in CHCl_3_, washed with saturated brines for 3 times. The crude product was purified by silica gel chromatography to give white powder. Colorless single crystals were grown from a methanol solution.

### Refinement

All H atoms were positioned geometrically and refined using a riding model, with d(C—H) = 0.95 - 0.99 Å, and *U*_iso_ (H) = 1.5 or 1.2 *U*_eq_(C).

### Crystal data

**Table:**

C_17_H_16_N_2_O_2_S	*F*(000) = 656
*M**_r_* = 312.38	*D*_x_ = 1.329 Mg m^−^^3^
Monoclinic, *P*2_1_/*n*	Cu *K*α radiation, λ = 1.54187 Å
Hall symbol: -P 2yn	Cell parameters from 2001 reflections
*a* = 14.174 (3) Å	θ = 27.6–72.2°
*b* = 5.9321 (12) Å	µ = 1.91 mm^−^^1^
*c* = 18.796 (4) Å	*T* = 113 K
β = 99.06 (3)°	Prism, colourless
*V* = 1560.7 (5) Å^3^	0.26 × 0.24 × 0.22 mm
*Z* = 4	

### Data collection

**Table:**

Rigaku Saturn diffractometer	3034 independent reflections
Radiation source: fine-focus sealed tube	2819 reflections with *I* > 2σ(*I*)
Multilayer monochromator	*R*_int_ = 0.045
Detector resolution: 14.63 pixels mm^-1^	θ_max_ = 72.5°, θ_min_ = 3.6°
ω scans	*h* = −17→17
Absorption correction: multi-scan (*CrystalClear*; Rigaku/MSC, 2005)	*k* = −7→7
*T*_min_ = 0.636, *T*_max_ = 0.678	*l* = −17→23
16000 measured reflections	

### Refinement

**Table:**

Refinement on *F*^2^	Secondary atom site location: difference Fourier map
Least-squares matrix: full	Hydrogen site location: inferred from neighbouring sites
*R*[*F*^2^ > 2σ(*F*^2^)] = 0.038	H-atom parameters constrained
*wR*(*F*^2^) = 0.098	*w* = 1/[σ^2^(*F*_o_^2^) + (0.0608*P*)^2^ + 0.3535*P*] where *P* = (*F*_o_^2^ + 2*F*_c_^2^)/3
*S* = 1.08	(Δ/σ)_max_ = 0.001
3034 reflections	Δρ_max_ = 0.30 e Å^−^^3^
201 parameters	Δρ_min_ = −0.28 e Å^−^^3^
1 restraint	Extinction correction: *SHELXL97* (Sheldrick, 2008), Fc^*^=kFc[1+0.001xFc^2^λ^3^/sin(2θ)]^-1/4^
Primary atom site location: structure-invariant direct methods	Extinction coefficient: 0.0040 (4)

### Special details

**Table:**

Geometry. All e.s.d.'s (except the e.s.d. in the dihedral angle between two l.s. planes) are estimated using the full covariance matrix. The cell e.s.d.'s are taken into account individually in the estimation of e.s.d.'s in distances, angles and torsion angles; correlations between e.s.d.'s in cell parameters are only used when they are defined by crystal symmetry. An approximate (isotropic) treatment of cell e.s.d.'s is used for estimating e.s.d.'s involving l.s. planes.
Refinement. Refinement of *F*^2^ against ALL reflections. The weighted *R*-factor *wR* and goodness of fit *S* are based on *F*^2^, conventional *R*-factors *R* are based on *F*, with *F* set to zero for negative *F*^2^. The threshold expression of *F*^2^ > σ(*F*^2^) is used only for calculating *R*-factors(gt) *etc*. and is not relevant to the choice of reflections for refinement. *R*-factors based on *F*^2^ are statistically about twice as large as those based on *F*, and *R*- factors based on ALL data will be even larger.

### Fractional atomic coordinates and isotropic or equivalent isotropic displacement parameters (Å^2^)

**Table:**

	*x*	*y*	*z*	*U*_iso_*/*U*_eq_	
S1	0.07455 (2)	0.16825 (5)	0.143145 (17)	0.02086 (13)	
O1	0.02603 (6)	−0.21422 (16)	0.06058 (5)	0.0237 (2)	
O2	−0.09148 (7)	−0.05922 (19)	0.11147 (6)	0.0356 (3)	
N1	0.38740 (7)	0.30018 (18)	0.16585 (6)	0.0202 (3)	
N2	0.64150 (12)	0.3514 (3)	0.02028 (9)	0.0527 (4)	
C1	0.09324 (9)	−0.0540 (2)	0.08710 (7)	0.0201 (3)	
C2	0.18393 (9)	−0.0624 (2)	0.07280 (7)	0.0201 (3)	
H2	0.2064	−0.1715	0.0425	0.024*	
C3	0.24163 (9)	0.1132 (2)	0.10896 (7)	0.0187 (3)	
C4	0.19251 (9)	0.2503 (2)	0.14809 (7)	0.0196 (3)	
C5	0.23440 (9)	0.4496 (2)	0.19049 (7)	0.0216 (3)	
H5A	0.2447	0.4142	0.2426	0.026*	
H5B	0.1904	0.5799	0.1820	0.026*	
C6	0.32936 (9)	0.5055 (2)	0.16606 (7)	0.0217 (3)	
H6A	0.3175	0.5712	0.1170	0.026*	
H6B	0.3643	0.6183	0.1990	0.026*	
C7	0.34597 (9)	0.1510 (2)	0.10650 (7)	0.0206 (3)	
H7A	0.3800	0.0047	0.1106	0.025*	
H7B	0.3537	0.2201	0.0598	0.025*	
C8	0.48585 (9)	0.3588 (2)	0.15919 (8)	0.0245 (3)	
H8A	0.5078	0.4827	0.1931	0.029*	
H8B	0.4879	0.4140	0.1097	0.029*	
C9	0.55298 (9)	0.1608 (2)	0.17475 (7)	0.0207 (3)	
C10	0.62212 (9)	0.1119 (2)	0.13184 (8)	0.0255 (3)	
C11	0.68601 (10)	−0.0673 (3)	0.14838 (9)	0.0321 (3)	
H11	0.7327	−0.0980	0.1185	0.038*	
C12	0.68115 (10)	−0.1995 (2)	0.20814 (9)	0.0311 (3)	
H12	0.7242	−0.3218	0.2195	0.037*	
C13	0.61310 (10)	−0.1526 (2)	0.25154 (8)	0.0276 (3)	
H13	0.6097	−0.2425	0.2929	0.033*	
C14	0.54986 (9)	0.0252 (2)	0.23487 (8)	0.0250 (3)	
H14	0.5035	0.0551	0.2651	0.030*	
C15	0.63104 (11)	0.2481 (3)	0.06926 (9)	0.0351 (4)	
C16	−0.06452 (9)	−0.2078 (2)	0.07641 (8)	0.0233 (3)	
C17	−0.12011 (10)	−0.4073 (2)	0.04513 (8)	0.0270 (3)	
H17A	−0.1192	−0.4134	−0.0069	0.040*	
H17B	−0.1862	−0.3947	0.0539	0.040*	
H17C	−0.0914	−0.5451	0.0678	0.040*	

### Atomic displacement parameters (Å^2^)

**Table:**

	*U*^11^	*U*^22^	*U*^33^	*U*^12^	*U*^13^	*U*^23^
S1	0.01803 (19)	0.0246 (2)	0.0204 (2)	0.00246 (11)	0.00426 (13)	−0.00427 (11)
O1	0.0176 (5)	0.0254 (5)	0.0282 (5)	−0.0002 (4)	0.0038 (4)	−0.0072 (4)
O2	0.0237 (5)	0.0417 (6)	0.0437 (7)	−0.0034 (4)	0.0123 (5)	−0.0190 (5)
N1	0.0178 (5)	0.0186 (5)	0.0246 (6)	0.0000 (4)	0.0041 (4)	0.0001 (4)
N2	0.0543 (10)	0.0658 (11)	0.0433 (9)	0.0074 (8)	0.0242 (8)	0.0159 (8)
C1	0.0199 (6)	0.0217 (6)	0.0185 (6)	0.0014 (5)	0.0017 (5)	−0.0015 (5)
C2	0.0205 (6)	0.0205 (6)	0.0191 (6)	0.0041 (5)	0.0028 (5)	−0.0012 (5)
C3	0.0197 (6)	0.0200 (6)	0.0163 (6)	0.0024 (5)	0.0025 (5)	0.0022 (5)
C4	0.0191 (6)	0.0221 (6)	0.0174 (6)	0.0026 (5)	0.0028 (5)	0.0005 (5)
C5	0.0225 (6)	0.0216 (6)	0.0209 (7)	0.0025 (5)	0.0046 (5)	−0.0020 (5)
C6	0.0250 (6)	0.0179 (6)	0.0225 (7)	0.0009 (5)	0.0050 (5)	−0.0001 (5)
C7	0.0202 (6)	0.0202 (6)	0.0219 (7)	0.0014 (5)	0.0051 (5)	−0.0013 (5)
C8	0.0207 (6)	0.0224 (6)	0.0310 (8)	−0.0028 (5)	0.0063 (5)	0.0020 (5)
C9	0.0167 (6)	0.0218 (6)	0.0234 (7)	−0.0039 (5)	0.0020 (5)	−0.0015 (5)
C10	0.0214 (6)	0.0303 (7)	0.0255 (7)	−0.0028 (5)	0.0059 (5)	−0.0009 (6)
C11	0.0236 (7)	0.0369 (8)	0.0376 (9)	0.0032 (6)	0.0103 (6)	−0.0024 (7)
C12	0.0217 (7)	0.0268 (7)	0.0433 (9)	0.0026 (5)	0.0009 (6)	0.0013 (6)
C13	0.0242 (7)	0.0268 (7)	0.0303 (8)	−0.0040 (5)	0.0003 (6)	0.0052 (6)
C14	0.0226 (6)	0.0275 (7)	0.0257 (7)	−0.0023 (5)	0.0058 (5)	0.0011 (6)
C15	0.0322 (8)	0.0425 (9)	0.0337 (8)	0.0026 (7)	0.0152 (7)	0.0033 (7)
C16	0.0182 (6)	0.0285 (7)	0.0231 (7)	0.0011 (5)	0.0028 (5)	−0.0003 (5)
C17	0.0229 (7)	0.0270 (7)	0.0306 (8)	−0.0010 (5)	0.0029 (6)	−0.0025 (6)

### Geometric parameters (Å, º)

**Table:**

S1—C4	1.7297 (13)	C7—H7A	0.9900
S1—C1	1.7338 (13)	C7—H7B	0.9900
O1—C16	1.3629 (16)	C8—C9	1.5109 (18)
O1—C1	1.3819 (16)	C8—H8A	0.9900
O2—C16	1.1987 (17)	C8—H8B	0.9900
N1—C8	1.4625 (16)	C9—C14	1.393 (2)
N1—C6	1.4702 (16)	C9—C10	1.394 (2)
N1—C7	1.4716 (17)	C10—C11	1.399 (2)
N2—C15	1.135 (2)	C10—C15	1.449 (2)
C1—C2	1.3549 (18)	C11—C12	1.381 (2)
C2—C3	1.4284 (18)	C11—H11	0.9500
C2—H2	0.9500	C12—C13	1.386 (2)
C3—C4	1.3594 (18)	C12—H12	0.9500
C3—C7	1.5037 (17)	C13—C14	1.388 (2)
C4—C5	1.4955 (18)	C13—H13	0.9500
C5—C6	1.5256 (18)	C14—H14	0.9500
C5—H5A	0.9900	C16—C17	1.4905 (19)
C5—H5B	0.9900	C17—H17A	0.9800
C6—H6A	0.9900	C17—H17B	0.9800
C6—H6B	0.9900	C17—H17C	0.9800
			
C4—S1—C1	90.43 (6)	N1—C8—C9	112.25 (10)
C16—O1—C1	121.43 (10)	N1—C8—H8A	109.2
C8—N1—C6	110.18 (10)	C9—C8—H8A	109.2
C8—N1—C7	110.52 (10)	N1—C8—H8B	109.2
C6—N1—C7	110.09 (10)	C9—C8—H8B	109.2
C2—C1—O1	121.66 (11)	H8A—C8—H8B	107.9
C2—C1—S1	112.89 (10)	C14—C9—C10	117.63 (12)
O1—C1—S1	125.42 (9)	C14—C9—C8	120.42 (12)
C1—C2—C3	111.65 (11)	C10—C9—C8	121.90 (12)
C1—C2—H2	124.2	C9—C10—C11	121.26 (13)
C3—C2—H2	124.2	C9—C10—C15	120.83 (13)
C4—C3—C2	112.94 (11)	C11—C10—C15	117.90 (13)
C4—C3—C7	121.15 (12)	C12—C11—C10	119.92 (13)
C2—C3—C7	125.90 (11)	C12—C11—H11	120.0
C3—C4—C5	124.54 (12)	C10—C11—H11	120.0
C3—C4—S1	112.08 (10)	C11—C12—C13	119.56 (14)
C5—C4—S1	123.38 (9)	C11—C12—H12	120.2
C4—C5—C6	107.86 (10)	C13—C12—H12	120.2
C4—C5—H5A	110.1	C12—C13—C14	120.29 (14)
C6—C5—H5A	110.1	C12—C13—H13	119.9
C4—C5—H5B	110.1	C14—C13—H13	119.9
C6—C5—H5B	110.1	C13—C14—C9	121.34 (13)
H5A—C5—H5B	108.4	C13—C14—H14	119.3
N1—C6—C5	109.95 (10)	C9—C14—H14	119.3
N1—C6—H6A	109.7	N2—C15—C10	177.32 (17)
C5—C6—H6A	109.7	O2—C16—O1	122.22 (12)
N1—C6—H6B	109.7	O2—C16—C17	127.33 (12)
C5—C6—H6B	109.7	O1—C16—C17	110.45 (11)
H6A—C6—H6B	108.2	C16—C17—H17A	109.5
N1—C7—C3	110.09 (10)	C16—C17—H17B	109.5
N1—C7—H7A	109.6	H17A—C17—H17B	109.5
C3—C7—H7A	109.6	C16—C17—H17C	109.5
N1—C7—H7B	109.6	H17A—C17—H17C	109.5
C3—C7—H7B	109.6	H17B—C17—H17C	109.5
H7A—C7—H7B	108.2		
			
C16—O1—C1—C2	178.55 (12)	C4—C3—C7—N1	16.65 (17)
C16—O1—C1—S1	0.90 (18)	C2—C3—C7—N1	−163.09 (12)
C4—S1—C1—C2	−0.56 (11)	C6—N1—C8—C9	167.62 (11)
C4—S1—C1—O1	177.27 (12)	C7—N1—C8—C9	−70.50 (14)
O1—C1—C2—C3	−176.86 (11)	N1—C8—C9—C14	−46.34 (17)
S1—C1—C2—C3	1.06 (14)	N1—C8—C9—C10	136.16 (13)
C1—C2—C3—C4	−1.16 (16)	C14—C9—C10—C11	0.2 (2)
C1—C2—C3—C7	178.60 (12)	C8—C9—C10—C11	177.72 (13)
C2—C3—C4—C5	−179.07 (12)	C14—C9—C10—C15	−178.76 (13)
C7—C3—C4—C5	1.2 (2)	C8—C9—C10—C15	−1.2 (2)
C2—C3—C4—S1	0.74 (15)	C9—C10—C11—C12	0.0 (2)
C7—C3—C4—S1	−179.03 (10)	C15—C10—C11—C12	178.99 (14)
C1—S1—C4—C3	−0.12 (11)	C10—C11—C12—C13	−0.3 (2)
C1—S1—C4—C5	179.69 (11)	C11—C12—C13—C14	0.4 (2)
C3—C4—C5—C6	14.85 (18)	C12—C13—C14—C9	−0.2 (2)
S1—C4—C5—C6	−164.94 (10)	C10—C9—C14—C13	−0.1 (2)
C8—N1—C6—C5	−166.73 (11)	C8—C9—C14—C13	−177.70 (12)
C7—N1—C6—C5	71.13 (13)	C9—C10—C15—N2	162 (4)
C4—C5—C6—N1	−49.11 (14)	C11—C10—C15—N2	−17 (4)
C8—N1—C7—C3	−173.55 (10)	C1—O1—C16—O2	3.0 (2)
C6—N1—C7—C3	−51.61 (13)	C1—O1—C16—C17	−176.31 (11)

### Hydrogen-bond geometry (Å, º)

**Table:**

*D*—H···*A*	*D*—H	H···*A*	*D*···*A*	*D*—H···*A*
C11—H11···O2^i^	0.95	2.53	3.3346 (19)	143

Symmetry code: (i) *x*+1, *y*, *z*.
